# First person – Paco López-Cuevas

**DOI:** 10.1242/dmm.048972

**Published:** 2021-03-19

## Abstract

First Person is a series of interviews with the first authors of a selection of papers published in Disease Models & Mechanisms, helping early-career researchers promote themselves alongside their papers. Paco López-Cuevas is first author on ‘Transformed notochordal cells trigger chronic wounds in zebrafish, destabilizing the vertebral column and bone homeostasis’, published in DMM. Paco is a PhD student in the lab of Paul Martin at the University of Bristol, Bristol, UK, investigating the role of inflammation in cancer progression.


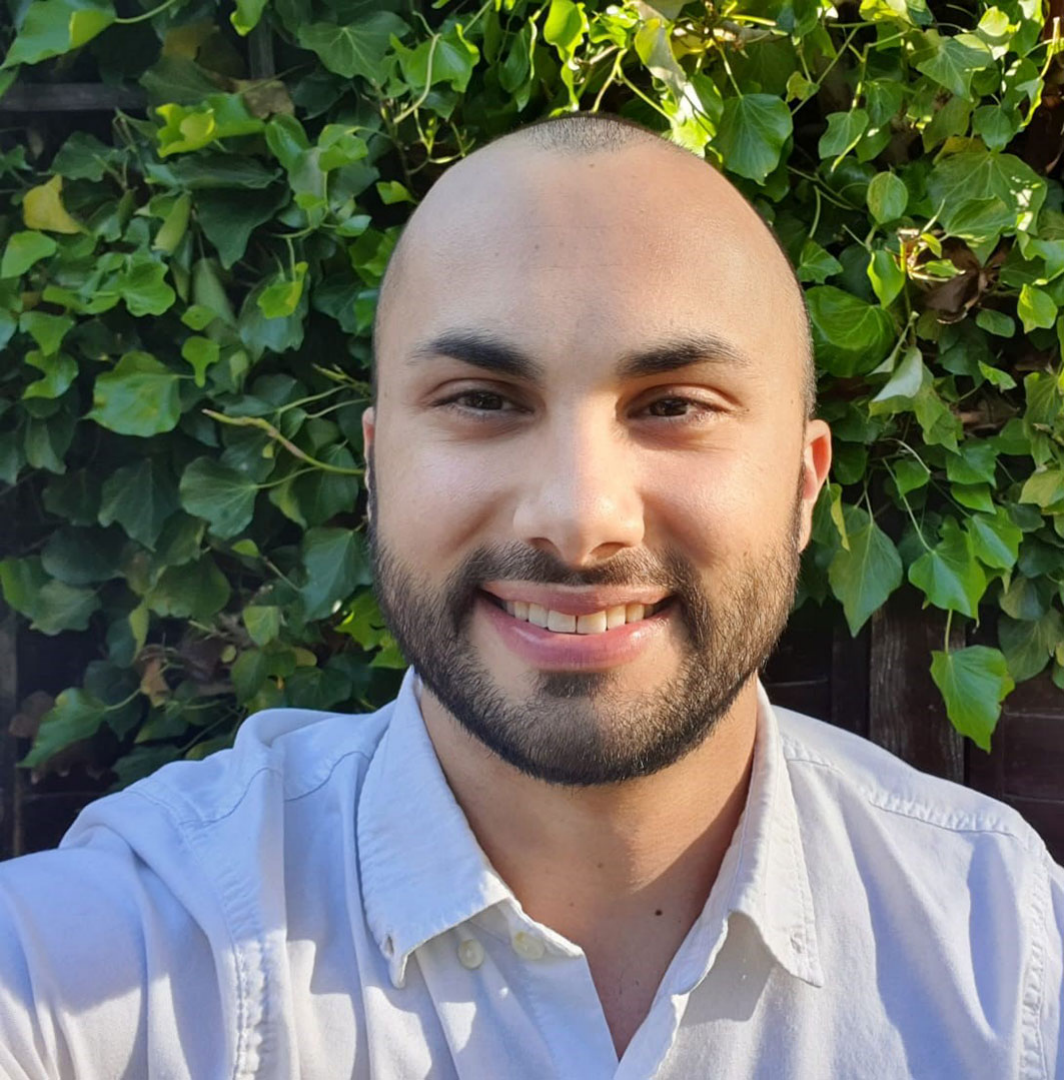


**Paco López-Cuevas**

**How would you explain the main findings of your paper to non-scientific family and friends?**

Chordoma is a rare type of bone cancer, affecting approximately one individual per million every year. This cancer can compromise different locations of the spine, but often appears in the base of the skull, where surgeries are challenging. Chordomas originate from internal cells of the spine known as notochord cells, which form part of the notochord. The notochord plays an important role in the formation of the spine, but when notochord cells get transformed into cancerous cells, they grow and divide uncontrollably, and this can result in chordomas. To study the impact of notochord cancer cells in spine formation and bone quality, we used a zebrafish line that induces cancer in the notochord cells. Interestingly, we saw that the notochord cells of these fish divide faster than normal, and they induce a local wound-like response, characterised by increased inflammation and alterations in the sheath layer of collagen that wraps the notochord. These changes have an impact on how the spine is formed in these fish, resulting in vertebral defects that we term butterfly vertebrae, because they look like they have ‘wings’. We also saw an imbalance between the cells that form bone (osteoblasts) and those that destroy bone (osteoclasts), resulting in poor-quality bones. The coolest observation was that when we get rid of inflammatory cells, the notochord cancer cells stop dividing uncontrollably, and fish have fewer butterfly vertebrae. Eureka! We might have found a solution for chordoma in our fish model by playing with the number of inflammatory cells, and this could be a potential way to treat chordomas in human.

“[…] when we get rid of inflammatory cells, the notochord cancer cells stop dividing uncontrollably, and fish have fewer butterfly vertebrae.”

**What are the potential implications of these results for your field of research?**

Our results suggest that the zebrafish chordoma model used here could be an important tool to help us better understand chordoma biology and how transformed notochord cells and inflammation could be involved in the development of spine malformations and malignancy. Our findings on the role of inflammatory innate immune cells in the development of notochord lesions and subsequent vertebral defects suggest that inflammation could be a promising pharmacological target for chordoma. Overall, our study supports parallels between chordoma and wound-triggered inflammation.

**What are the main advantages and drawbacks of the model system you have used as it relates to the disease you are investigating?**

We used a zebrafish cancer model in our studies. Zebrafish are teleost fish that have emerged as advantageous animal models to study a variety of human diseases, including bone diseases and cancers, due to several reasons such as their fast development, translucency and feasible genetic manipulation. Their translucency allows time-lapse confocal imaging to study cell behaviour in real time, in a non-invasive way and without the need to sacrifice animals, something that would not be possible using other animal models. In addition, during the first weeks of development, zebrafish do not have a functional adaptive immune system, allowing the investigation of the innate immune response, on its own. All of these advantages, together with the availability of reporter lines labelling specific cell types, the feasibility of generating mutants for different genes and their skeletal similarities to humans, led us to choose zebrafish to study chordoma. More specifically, a major advantage of our zebrafish chordoma model is the fish survival to adulthood. While previously reported zebrafish chordoma models were lethal at larval stages, our model allowed us to analyse changes from developmental stages to bone homeostasis in adult zebrafish, using techniques such as micro-computed tomography (CT) and histological analysis. Bone changes are characteristic of human chordomas; therefore, our model would be suitable to study aspects of chordoma not possible before, adding the possibility of testing new drugs for therapeutic applications in chordoma development and bone maintenance.

A limitation of our model is that transformation of notochord cells is achieved by expression of a mutated RAS gene, which is not commonly observed in chordoma patients. However, our RAS model mimics the downstream signalling driven by the activation of epidermal growth factor receptor (EGFR), which is well known to be highly expressed in human chordomas. Another drawback of our model is the fact that transformation is not only induced in notochord cells, but also in melanoblasts, resulting in melanoma development. Fortunately, this can be avoided by combination with available pigment-free fish lines.

**What has surprised you the most while conducting your research?**

During the development of this research, we read in the literature that the progression of several cancers could be mediated by an inflammatory response, and that inhibition of inflammatory cells might be a desirable treatment to prevent cancer growth. We were really surprised when we found out in our own hands that chordoma is a type of cancer that could be treated by depletion of inflammatory cells. This finding was a very striking result that really motivated us to carry on with our investigation. Another surprising moment was when we realized that our zebrafish chordoma model had extensively been used by others to study melanoma for many years (these fish also express oncogenic RAS in melanoblasts, thus modelling melanoma), but no one had previously paid attention to their skeletal changes.

**Describe what you think is the most significant challenge impacting your research at this time and how will this be addressed over the next 10 years?**

Chordomas are highly resistant to current mainstream cancer treatments, including chemotherapy and radiotherapy, which is the reason why surgery is the main strategy to treat this type of cancer. However, due to the proximity of chordomas to vital structures (e.g. nerves or blood vessels), surgery remains challenging and often fails to achieve a complete removal of the tumour, increasing the probability of cancer recurrence. Therefore, alternative interventions are needed for chordoma patients. In our study, we report that immunotherapy, in particular the suppression of innate inflammatory cells, could be a suitable therapeutic approach for chordoma. Due to its success, this type of treatment modality has increased in popularity over the last years, especially among those cancers where inflammation clearly benefits cancer growth. While immunotherapy is very promising, further research will be required, and animal models like the zebrafish will undoubtedly be valuable to extrapolate results to the clinic.

**Figure:**
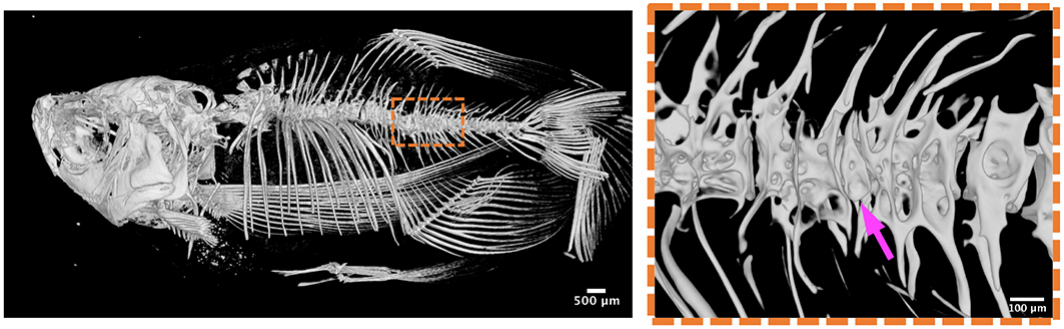
**Micro-CT images of adult zebrafish (6 months) showing vertebral column fusions (arrow) in our chordoma model.**

**What changes do you think could improve the professional lives of early-career scientists?**

In my case, after I finished my undergraduate studies, I had the opportunity to conduct a research internship abroad. This helped me to network and establish new collaborations, allowing me to grow scientifically and ultimately facilitating the possibility to start my PhD studies. Being exposed to a completely different laboratory environment outside my country and make new connections was definitely key in the beginning of my scientific career. Therefore, I believe that early professionals would benefit from more funding schemes that are specifically dedicated to this matter. In addition, the election of the laboratory where you would like to carry out your PhD is a fundamental element that can strongly influence your future scientific career. In this line, although I am aware that several universities have begun to do this, I would encourage the implementation of PhD programmes with rotations or short internships in different laboratories at the beginning of these studies and prior to the final laboratory election. This would allow PhD students to get to know their potential PhD supervisor and work with laboratory members, helping them in their decision before this long journey starts.

During my PhD, I have been very fortunate to have Prof. Paul Martin as my PhD supervisor. He has always encouraged me to establish collaborations with other scientists outside our laboratory. For instance, this study has been the result of a collaborative work with Dr Erika Kague, who has also been a great mentor for me and has taught me a lot about the bone field. Therefore, in my view, scientists should collaborate from very early in their careers, not only because they could learn from other mentors different from their principal investigators, but it could also increase their chances to publish in scientific journals.

Some early-career scientists choose to leave academia due to the uncertainty of this field. Offering permanent postdoctoral positions to those who are not interested in becoming a principal investigator or lecturer, but they rather prefer to perform experiments in the laboratory, would increase the number of scientists remaining in their research jobs.

“[…] the election of the laboratory where you would like to carry out your PhD is a fundamental element that can strongly influence your future scientific career.”

**What motivated you to pursue a scientific career and what are your next steps?**

Since I started my undergraduate studies, I have been interested in research, so I exposed myself to different laboratories where I enjoyed designing and executing experimental approaches to try and give an answer to biological questions, particularly those that were associated with human pathologies. However, it was not until my first research internship abroad mentioned above that I could contextualize the social, clinical and economic impact that my role as a biomedical researcher could have. My goal during this internship was to investigate new approaches for tuberculosis treatment using the zebrafish model. Seeing that results of my own experiments could have a clinical application was very motivating and made me realise the personal and professional satisfaction that research can create on me. That was the turning point when I decided that I want to pursue a biomedical scientific career, with the hope that I can contribute to our current knowledge of human diseases and help to develop new treatment strategies. Because I am particularly interested in the study of cancer and inflammation, after completion of my PhD I would like to combine all my previous expertise to continue my research in this field and start working as a postdoctoral fellow.
